# Consciousness Research Through Pain

**DOI:** 10.3390/healthcare13030332

**Published:** 2025-02-06

**Authors:** Dong Ah Shin, Min Cheol Chang

**Affiliations:** 1 Department of Neurosurgery, Spine and Spinal Cord Institute, Severance Hospital, Yonsei University College of Medicine, 50-1, Yonsei-ro, Seodaemun-gu, Seoul 03722, Republic of Korea; cistern@yuhs.ac; 2 Department of Physical Medicine and Rehabilitation, College of Medicine, Yeungnam University, 170 Hyeonchung-ro, Nam-gu, Daegu 42415, Republic of Korea

**Keywords:** consciousness, pain, research, neural network, mental health

## Abstract

**Background/Objectives:** Consciousness is a complex and elusive phenomenon encompassing self-awareness, sensory perception, emotions, and cognition. Despite significant advances in neuroscience, understanding the neural mechanisms underlying consciousness remains challenging. Pain, as a subjective and multifaceted experience, offers a unique lens for exploring consciousness by integrating sensory inputs with emotional and cognitive dimensions. This study examines the relationship between consciousness and pain, highlighting the potential of pain as a model for understanding the interplay between subjective experience and neural activity. **Methods:** Literature review. **Results:** Key theories of consciousness, such as the Global Workspace Theory and the Integrated Information Theory, provide diverse frameworks for interpreting the emergence of consciousness. Similarly, pain research emphasizes the role of subjective interpretation and emotional context in shaping sensory experiences, reflecting broader challenges in consciousness studies. The limitations of current methodologies, particularly the difficulty of objectively measuring subjective phenomena, like pain and consciousness, are also addressed. This highlights the importance of neural correlates, with a particular focus on brain regions, such as the anterior cingulate cortex and the insula, which bridge sensory and emotional experiences. By analyzing the shared attributes of pain and consciousness, this study underscores the potential for pain to serve as a measurable proxy in consciousness research. **Conclusions:** Ultimately, it contributes to unraveling the neural and philosophical underpinnings of consciousness, offering implications for mental health treatment and advancements in artificial intelligence. This study fills a critical gap by leveraging pain as a measurable and reproducible model for exploring the neural and subjective mechanisms of consciousness. By combining theoretical frameworks with empirical evidence, it offers novel insights into how consciousness emerges from neural processes.

## 1. Introduction

Consciousness is a profound and elusive phenomenon within the realm of scientific inquiry [1]. Broadly defined, it encompasses the ability to perceive oneself and the environment by integrating sensory experiences, thoughts, emotions, and self-awareness [2]. Despite significant advancements in neuroscience and cognitive science, the precise mechanisms underlying consciousness remain unclear. Understanding consciousness is vital not only for advancing knowledge of the human mind but also for improving treatments for mental health disorders, such as depression and anxiety [3].

Pain, a complex and subjective experience, extends beyond simple sensory perception to include emotional and cognitive components [4]. Pain research provides a valuable opportunity to explore the nature of consciousness itself [5,6]. By investigating how the brain perceives and interprets pain, we can gain deeper insights into the subjective nature of experience and the neural processes that give rise to consciousness [5,6].

In this study, we explore the relationship between consciousness and pain, examining how pain, as a conscious experience, can offer unique insights into broader questions about how and why we perceive the world as we do. The study delves into the features of consciousness, the challenges of studying it, and several prominent theories explaining its mechanisms. Additionally, we highlight the critical role pain plays in consciousness research, demonstrating how pain, as both a sensory and emotional experience, serves as a model for understanding the neural and subjective aspects of consciousness. By integrating pain research with broader studies on consciousness, we can address the central mystery of human experience: the connection between the mind and the brain. Understanding the intricate relationship between pain and consciousness is essential for bridging the gap between subjective experiences and their neural correlates. This study uniquely addresses this need by exploring how pain serves as a measurable and reproducible model to understand consciousness, offering new avenues for theoretical insights and practical applications.

## 2. What Is Consciousness?

Consciousness is defined differently across various fields, but it generally refers to the ability to perceive oneself and the surrounding environment [2,7]. It is a complex phenomenon encompassing a wide range of subjective experiences, including thoughts, emotions, sensations, and pain [8]. Consciousness enables us to understand and respond to current situations by integrating external sensory information with internal thoughts, emotions, and memories [7]. It also plays a crucial role in evaluating situations and making behavioral choices [9]. Moreover, consciousness allows individuals to be aware of their thoughts and feelings; in other words, it facilitates self-awareness [2]. Without consciousness, humans would be limited to basic reflexive responses.

Consciousness research has the potential to make significant contributions across multiple fields. Understanding consciousness could lead to better identification of the causes of and treatments for mental health disorders [3]. For instance, depression and anxiety are closely linked to the emotional aspects of consciousness. Additionally, studying consciousness is essential for exploring the relationship between the brain and behavior. Researchers can deepen their understanding of brain functions by examining how different brain regions contribute to conscious experience [10]. Consciousness research also holds implications for the development of artificial intelligence (AI) [11,12]. If we can comprehend human cognition and the essential nature of consciousness, it might become possible to move beyond general AI and create self-aware systems capable of experiencing emotions. Furthermore, understanding consciousness could enhance our ability to empathize with others by providing deeper insights into their experiences and emotions, thereby improving social interactions. Ultimately, these advancements are fundamental to understanding human life and addressing numerous societal challenges.

## 3. Features of Consciousness

There are ten key features of consciousness. The first is subjectivity. Consciousness is inherently a personal experience [13]; each person perceives the world uniquely, making it impossible to perfectly share or understand another person’s experiences, including pain, emotions, and thoughts. The second feature of consciousness is continuity, characterized by an ever-changing yet continuous stream of experiences [14]. While each moment is new, these experiences are interconnected, creating a consistent sense of self-awareness. The third key feature is self-awareness, or the ability to perceive oneself [15,16]. Beyond perceiving external stimuli, individuals are aware of their thoughts, feelings, and reactions to these stimuli. The fourth feature is intentionality, wherein consciousness is always directed toward an object, entity, or event [17]; it cannot simply exist in an “empty” state and continually involves focused experiences. The fifth feature is selective attention. Because the amount of information that can be processed simultaneously is limited, consciousness selectively focuses on salient information or stimuli [18], while other information is processed subconsciously or ignored. The sixth feature of consciousness is that it is accompanied by qualia, the subjective sensory experience of a given stimulus [19]. Qualia represents the qualitative [19] and nuanced aspect of individual experiences. The seventh feature is plasticity, because consciousness is highly fluid and adaptable [20]. For example, different states of consciousness, such as dreaming, hypnosis, and drug-induced states, can alter their content and nature [21]. The eighth feature is temporal awareness, which is the ability to perceive the flow of time [22]. Humans perceive the present, recall the past, and plan for the future, organizing experiences into a coherent temporal framework. The ninth feature is unity of consciousness, whereby multiple sensory inputs are integrated to form a single, consistent experience [23]. Sensations like vision, hearing, and touch combine to create a cohesive perception of the world. Finally, consciousness extends beyond sensory experiences to include functions, such as planning, decision making, and behavior control [24]. This aspect, often referred to as executive function, is central to goal-oriented behavior in humans.

## 4. Difficulties in Consciousness Research

Chalmers, a prominent advocate of dualism, has argued that physicalist explanations of consciousness are insufficient. He posits that the subjective aspects of consciousness, such as qualia, cannot fully be reduced to physical processes [25]. Chalmers emphasizes that consciousness is essentially subjective; that is, individual experiences are unique and cannot be directly observed or measured [25]. This subjectivity poses significant challenges for scientific methodologies for studying consciousness [26]. A conscious experience is inherently opaque to external observers and, therefore, difficult to verify or measure experimentally. Nagel has posed fundamental questions about the nature of experience, asking, in particular, what it feels like to “be” something [27]. He argues that humans could never fully comprehend a bat’s experience of perceiving the world through sound waves, underscoring the idea that the subjective nature of conscious experience cannot be reduced to purely physical explanations [27]. Levine has introduced the concept of the “explanatory gap”, highlighting the lack of physical explanations that bridge the process through which neural activity gives rise to conscious experience [28]. He emphasizes that this gap indicates the insufficiency of physicalist theories in addressing the subjective aspects of consciousness [28]. Pain research illustrates a similar challenge. The subjective nature of pain means that its assessment relies entirely on self-reported expressions [29] and cannot be objectively measured. External pain is difficult to evaluate using an experimental approach. Currently, there are no objective tools or indices for measuring consciousness or pain. Techniques that measure brain activity, such as EEG and fMRI, can show patterns of brain activation, but it is unclear how these patterns are related to specific experiences of consciousness or pain [30,31]. For example, increased brain activity in a particular region does not necessarily prove that the activity directly corresponds to a conscious experience. Despite studies identifying specific brain regions or neural circuits as critical for consciousness, the mechanism through which neural activity generates conscious experience remains a major puzzle [32,33,34]. Koch has argued that studying the neural correlates of consciousness (NCC) is essential for understanding the phenomenon [35]. He viewed consciousness as the product of specific brain regions or circuits and considered the occipital cortex to play an important role in consciousness. However, no study has been able to accurately identify NCC. McGinn has further posited that humans may lack the cognitive tools required to fully explain consciousness, making research in this field especially challenging [36].

Furthermore, locating the neural origins of consciousness remains a significant challenge, and anesthesiology provides unique insights by observing how different states of consciousness are modulated during anesthesia [37]. Unresponsiveness does not always equate to the absence of consciousness or connectedness [37]. This underscores the complexity of detecting and monitoring consciousness. Advances in anesthesia research hold the potential for developing reliable tools to identify distinct consciousness states, ultimately improving patient management and contributing to theoretical models of consciousness.

## 5. Theories of Consciousness

In this section, we introduce various theories proposed to explain the phenomenon of consciousness (Table 1).

### 5.1. Global Workspace Theory (GWT)

The GWT posits that consciousness arises when information processed across various brain regions is unified and shared within a global workspace [38]. This workspace functions as a virtual stage where information is integrated and consciously processed through attention and cognitive mechanisms. Subconscious information remains inaccessible to conscious awareness until it enters this space. The GWT describes consciousness as a collaborative process that occurs at a single central stage, with attention and information integration playing important roles. However, while the GWT provides a framework for understanding how information might be unified, it does not address the existence or mechanisms of an internal observer (“homunculus”) that would perceive and experience this unified information.

### 5.2. The Attention Schema Theory (AST)

The AST suggests that consciousness arises from attention [39,40,41]. The focus of attention emerges as the content of consciousness. When attention is focused on a given object or event, that object is consciously perceived and processed. This theory views consciousness as the result of selective attention and explains that only certain information is consciously processed. This theory emphasizes that attention is the core element of consciousness and that the content of consciousness differs depending on how attention changes. Attention plays a vital role in pain management. Specifically, attention can cause pain to be perceived even more intensely; conversely, spreading one’s attention can reduce the sensation of pain.

### 5.3. Integrated Information Theory (IIT)

The IIT proposes that consciousness emerges from the integration of information within a system [42,43,44,45]. The more effectively a system integrates multiple sources of information for processing, the higher its level of consciousness. This theory emphasizes that integrated information gains a new quality greater than the sum of its components. In other words, consciousness arises when each piece of information does not exist independently but in a mutually unified form. The IIT is an effort to define consciousness mathematically and suggests that the amount of information integration performed by a given brain region or neural circuit can be measured to explain the level of consciousness. However, this theory only explains that consciousness arises from the information integration process and fails to explain how consciousness arises.

### 5.4. Higher-Order Theories

The Higher-Order Theories propose that conscious experience occurs when first-order sensory information is reflected in second-order perception (metacognition) [46]. For sensory information to become a part of conscious awareness, a higher-order perception of that sensory information is required. For example, becoming consciously aware of hearing a sound requires not only perceiving the sound but also recognizing the act of hearing it. Consciousness arises from the sensation itself and the cognitive evaluation of the sensation [47]. These theories explain that if there is no higher-order awareness, simple sensations and thoughts will not result in consciousness.

### 5.5. Predictive Processing Theory

The Predictive Processing Theory suggests that the brain is constantly making predictions about the environment, and consciousness emerges as a result of error correction in response to these predictions [48]. Rather than passively receiving environmental stimuli, the brain actively anticipates future stimuli. The brain endlessly predicts events occurring in the environment, compares these predictions with actual sensory inputs, and forms conscious experiences during this process. If the predictions are accurate, we perceive them consciously; if the predictions are incorrect, we consciously correct the errors. The processes of prediction and correction are emphasized as important mechanisms for consciousness, and brain predictions play a more important role than sensory inputs.

### 5.6. Enactive Theories of Consciousness

The Enactive Theories of Consciousness propose that consciousness is not solely the product of brain activity but arises from interactions between the body and the environment [49]. These theories argue that consciousness arises inside of the brain and is constituted by the interactions between the body and the environment. It has been claimed that physical experiences and interactions with the outside world create consciousness. In other words, consciousness does not arise from isolated neural activity but rather arises when we explore and act within the world. Enactive theories of consciousness are differentiated from conventional approaches because they do not restrict consciousness to only the brain but also consider the body and the environment as important constituents of consciousness.

### 5.7. Functionalism

Functionalism states that consciousness is defined by physical structures as well as functions performed by the brain [50]. According to this theory, consciousness does not depend on specific structures or physical properties of the brain but is rather defined by functional roles and methods of information processing. This means that any system—biological or artificial—that performs equivalent functions could possess consciousness. Functionalism emphasizes the functional characteristics of the brain over structural characteristics to understand consciousness and approaches the essential nature of consciousness from the perspective of information processing.

## 6. Commonalities Between Consciousness and Pain

Pain is an integral part of consciousness. It is a complex phenomenon that combines physical, emotional, and social elements, requiring consciousness to interpret and respond to pain beyond a simple sensory stimulus [51]. By controlling consciousness-related pain pathways, it should be possible to control the intensity and quality of pain [52]. Below, we discuss several important aspects of the role of consciousness in pain. As a nervous system response, pain is perceived and assessed through consciousness [53]. Conscious awareness allows humans to go beyond merely detecting stimuli; it enables them to evaluate whether a stimulus is painful, determine its intensity, and decide how to react [54]. Pain is not only a simple sensory experience but also accompanied by emotional elements [55]. Through memory, consciousness associates pain with emotions, such as fear or anxiety, creating a learning mechanism to avoid similar stimuli in the future [55]. Conscious thought processes also play an important role in pain management [56]. For example, conscious adjustments make diverting attention or reinterpreting pain possible. Furthermore, being mindful of our pain enables us to request help from others or receive social support. Humans can receive treatment or care by verbally expressing their experiences of pain through consciousness and sharing their experiences with others.

The study of consciousness is as enigmatic as unresolved problems in quantum mechanics. Despite extensive research, consciousness remains poorly understood. This field involves numerous hypotheses and is closely related to philosophy. When classifying the theories of consciousness, there is still an ongoing debate between dualism, which views consciousness and the brain as separate entities, and reductionism, which argues that consciousness arises solely from brain activity [57]. Research into consciousness faces significant obstacles. First, subjective experiences are inherently difficult to measure objectively. Second, the brain’s complex neural mechanisms complicate the analysis of conscious processes. Third, the diversity of philosophical definitions and theoretical approaches hampers consensus in the field [58]. There are no methods to directly measure consciousness, and existing brain imaging techniques only allow for indirect observation of neural activity. Consciousness remains an intractable scientific problem. Similarly, pain research encounters challenges due to the subjective nature of pain perception, making measurement and interpretation difficult [59]. The neural mechanisms of pain are complex, and pain sensation is perceived differently by each individual, making it difficult to objectively judge pain intensity characteristics through brain activity [60].

Both consciousness and pain research share methodological and conceptual difficulties. Diverse theoretical definitions of pain and consciousness hinder consistent study, yet pain research provides valuable insights for understanding consciousness [61]. For instance, the process of detecting and interpreting pain closely mirrors that of perceiving conscious experiences. By examining these parallels, pain research can contribute to uncovering the mechanisms and essence of consciousness [62].

## 7. Why Pain Is Important for Consciousness Research

Pain is not merely a bodily response to stimuli; it is a subjective experience that is consciously felt. It begins with physiological nociception, which detects physical injury but transcends the simple transmission of stimuli [63]. As pain information is processed in the brain, it incorporates psychiatric, emotional, and cognitive components [64]. Through this process, pain transforms into a distressing or unpleasant experience, extending beyond a mere sensory phenomenon [64].

The aversive experience of pain exemplifies how consciousness arises from the coordinated activation of neural networks [65]. The “pain matrix” illustrates this process, transitioning from unconscious sensory inputs to conscious pain through the interaction of the sensorimotor, limbic, and frontoparietal networks. Intracortical EEG studies reveal that this sequence begins in the sensory, motor, and limbic areas, progresses to the anterior insular and frontoparietal networks, and culminates in the posterior cingulate and medial temporal areas, integrating self-awareness and autobiographical memories [65]. These findings highlight how pain serves as a model for understanding the mechanisms of consciousness, particularly the dynamic integration required for conscious perception.

In addition, assessing and managing pain in patients with consciousness disorders are critical and complex components of care directly linked to detecting consciousness and facilitating recovery [66]. Behavioral tools, such as the Nociception Coma Scale and its revised version, have been developed to evaluate pain responses in patients with consciousness disorders, despite ongoing debates about their accuracy and reliability [66]. Recent studies indicate that precise pain assessments may provide valuable insights into changes in the level of consciousness, particularly in patients diagnosed with unresponsive wakefulness syndrome or a vegetative state.

Melzack et al. have emphasized that pain is not simply a peripheral nervous stimulus but involves integrative processing within the central nervous system [67]. This highlights the cognitive dimensions of pain and underscores its nature as a complex conscious experience, providing a foundation for exploring the interplay between pain and consciousness [67]. Similarly, Craig has conceptualized pain as an emotional experience linked to homeostasis, proposing that it integrates physiological states with emotional experiences [68]. By defining pain as a sensory–affective phenomenon, Craig suggests its pivotal role in understanding conscious experiences. Grahek has analyzed pain through subjective, objective, and intersubjective lenses, asserting that its subjective nature offers valuable insights into consciousness research [69]. He stresses that the subjective experience of pain could provide new insights into consciousness research. In consciousness research, pain is an important tool for studying the internal experiences of humans and interactions in the brain as it cognitively processes these experiences [70]. Ploner et al. examined a patient who had lost pain sensation yet still experienced the emotional distress associated with it [71]. Their findings illustrate that pain is not only a sensation but also closely related to the emotional aspects of conscious experience. This implies that pain experiences are central to consciousness research. To understand the neurological basis of pain, it is crucial to investigate the activation of networks of specific brain regions associated with consciousness. For example, painful experiences activate the frontal lobe, the insula, and the cingulate cortex, regions closely related to conscious experiences [70]. Rainville et al. used brain imaging to discover that the emotional elements of pain are processed in the anterior cingulate cortex [70]. This highlights that pain is more than a simple sensation; it is a subjective experience, including emotional and cognitive elements. Pain can be induced experimentally, and people can report their experiences, enabling researchers to correlate subjective experiences with objective neural activity. Patients or participants can describe the intensity and nature of pain by providing direct feedback on their conscious experiences. Individuals perceive pain differently depending on its intensity and location. Differences in pain thresholds or tolerance change depending on the state of consciousness, attention, and emotion, providing a useful model for studying how consciousness changes or is controlled by subjective experiences [72]. Dennett has analyzed subjective experiences, including pain, to explain various aspects of consciousness. He argues that pain transcends simple neural stimuli, encompassing cognitive elements, such as intentional stance, thus positioning it as a valuable tool for consciousness research [72].

Recent advancements in AI are also contributing to this field. AI-integrated systems are already being utilized for pain management, demonstrating the potential of AI to revolutionize assessment and treatment strategies [73,74]. Moreover, integrating somatometric data into AI algorithms offers a promising avenue for analyzing consciousness-related patterns, enabling more precise and dynamic pain and consciousness management in clinical settings [73,74].

## 8. Conclusions

The study of pain in consciousness research offers critical insights into the relationship between subjective experiences and neural activity. Pain is a complex phenomenon that combines sensory inputs with emotional experiences, enabling a more profound understanding of the nature and boundaries of consciousness. Pain provides a unique opportunity to compare self-reported experiences with objective neural data, suggesting its potential as a metric for consciousness research. Through its ability to link subjective experiences with measurable neural activity, pain serves as a pivotal model for advancing consciousness research. Future consciousness research, particularly in the context of pain, should integrate real-world data to bridge theoretical insights with practical applications. Such an approach can enhance the relevance of this interdisciplinary field and provide specific implications for advancing both theoretical understanding and clinical practices.

## Figures and Tables

**Table 1:** Summary of the theories of consciousness.

Theory	Main Concept	Key Points
Global Workspace Theory	Consciousness arises from unified information shared in a “global workspace”.	Integration and sharing of information are emphasized; the subconscious remains inaccessible without integration.
Attention Schema Theory	Consciousness results from focused attention on specific objects or events.	Selective attention is highlighted; shifts in attention alter one’s conscious experience.
Integrated Information Theory	Consciousness emerges from the integration of information within a system.	More integration equals higher consciousness; the theory focuses on information unity but lacks mechanistic explanation.
Higher-Order Theory	Consciousness requires second-order reflection on first-order sensory data.	Metacognition or higher-order awareness is crucial for conscious experience.
Predictive Processing Theory	The brain forms consciousness through prediction and error correction.	Consciousness evolves from actively anticipating and revising sensory inputs.
Enactive Theory	Consciousness arises from interactions between the body and the environment.	Consciousness is a result of embodied and interactive processes rather than isolated brain activity.
Functionalism	Consciousness is defined by the brain’s functional roles rather than its physical structures.	Artificial systems could exhibit consciousness if functional equivalence is achieved.

## Data Availability

No new data were created or analyzed in this study. Data sharing is not applicable to this article.
